# Association between the fasting blood glucose to high-density lipoprotein cholesterol ratio and non-alcoholic fatty liver disease in Chinese adults: a secondary analysis

**DOI:** 10.3389/fmed.2026.1887682

**Published:** 2026-10-08

**Authors:** Da Lin, Gengbin Lin, Zhe Liu

**Affiliations:** 1 Department of Gastroenterology, Jieyang People’s Hospital, Jieyang, Guangdong, China; 2 The Intensive Care Unit, Jieyang People’s Hospital, Jieyang, Guangdong, China

**Keywords:** Chinese, fasting blood glucose, high-density lipoprotein cholesterol, non-alcoholic fatty liver disease, ratio

## Abstract

**Background:**

Elevated fasting blood glucose (FBG) and decreased high-density lipoprotein cholesterol (HDL-C) are individually associated with non-alcoholic fatty liver disease (NAFLD). This study investigated whether their composite ratio (GHR) is independently associated with NAFLD and whether this association varies across subgroups.

**Methods:**

This secondary analysis included 1,592 Chinese adults (40–70 years) from routine health checks. NAFLD was diagnosed via ultrasound. GHR was calculated as FBG divided by HDL-C. Multivariable logistic regression assessed the GHR–NAFLD association. Receiver operating characteristic (ROC) curves compared the discriminative ability of GHR with that of FBG and HDL-C alone using DeLong's test. Subgroup and interaction analyses were performed.

**Results:**

NAFLD prevalence was 61.1%. After full adjustment, each 1-unit increase in GHR was associated with 66% higher NAFLD odds [odds ratio (OR) = 1.66, 95% confidence interval (CI): 1.02–2.69]. Across quartiles, a significant positive trend persisted (*p* for trend = 0.017). Compared with the lowest quartile, the fully adjusted ORs were 1.36 (*p* = 0.076) for quartile 2, 1.73 (95% CI: 1.16–2.60, *p* = 0.008) for quartile 3, and 1.74 (95% CI: 1.03–2.93, *p* = 0.037) for quartile 4. In exploratory subgroup analyses, a borderline interaction with diabetes was observed (*P* for interaction = 0.046). GHR was associated with NAFLD in non-diabetic participants (OR = 2.17, 95% CI: 1.13–4.18) but not in diabetic participants. The AUC for GHR (0.684) exceeded that of FBG (0.640, *p* = 0.001) and HDL-C (0.637, *p* < 0.001). The fully adjusted model achieved an AUC of 0.799 (95% CI: 0.777–0.821).

**Conclusion:**

GHR was independently associated with NAFLD and showed better discrimination than FBG or HDL-C alone. However, adding GHR to the fully adjusted model did not significantly improve discrimination, and the possible stronger association in non-diabetic individuals requires confirmation. Its incremental value beyond established clinical risk factors is therefore limited, and GHR should not replace comprehensive risk assessment.

## 1 Introduction

Non-alcoholic fatty liver disease (NAFLD) has emerged as the most prevalent chronic liver disease worldwide, affecting an estimated 32.4% of the global population (1). The disease burden of NAFLD has increased by approximately 50.4% over the past three decades (2), posing a significant challenge to global public health (3). In China, the prevalence of NAFLD among adults has reached approximately 29.6%, with a notable rising trend (4). NAFLD encompasses a spectrum of liver pathologies ranging from simple hepatic steatosis to non-alcoholic steatohepatitis (NASH), which may progress to cirrhosis and hepatocellular carcinoma (5, 6).

The pathogenesis of NAFLD is complex and closely intertwined with metabolic dysregulation (7). Insulin resistance is widely recognized as a central driver of NAFLD, promoting hepatic *de novo* lipogenesis, impairing fatty acid oxidation, and altering lipoprotein metabolism (8, 9). Dyslipidemia, characterized by elevated triglycerides, increased low-density lipoprotein cholesterol, and decreased high-density lipoprotein cholesterol (HDL-C), is frequently observed in patients with NAFLD (10, 11). Reduced HDL-C levels are associated with impaired cholesterol efflux capacity and qualitative changes in HDL particles in NAFLD patients (12). Concurrently, elevated fasting blood glucose (FBG) levels, reflecting glucose metabolic dysfunction, are also strongly associated with NAFLD (13).

Given that both elevated FBG and decreased HDL-C are independently associated with NAFLD, it has been hypothesized that the ratio of fasting blood glucose to high-density lipoprotein cholesterol (GHR) may serve as a composite indicator that integrates these two metabolic abnormalities and provides better discriminative ability for NAFLD. A population-based analysis of American adults found that higher GHR was independently associated with both NAFLD prevalence and liver steatosis severity, with an area under the curve (AUC) of 0.731 for identifying NAFLD (14). Another retrospective study reported that FHR exhibited a non-linear relationship with NAFLD and significantly outperformed FBG or HDL-C alone in identifying affected individuals (15). Despite these promising findings, several gaps remain. First, data from Chinese populations are scarce, and it is unclear whether the GHR-NAFLD relationship persists after rigorous adjustment for body composition indices such as the fat-to-muscle ratio (FMR), which is strongly linked to steatosis (16). Second, whether metabolic comorbidities—particularly diabetes—modify the association between GHR and NAFLD or its discriminative performance of GHR has not been adequately examined. Third, most prior studies focused on the crude discriminative ability of GHR; few have evaluated how its discriminative performance changes after sequential adjustment for confounders, a step critical to understanding its independent contribution.

Using a publicly available dataset from Chinese health-checkup participants, we sought to determine whether GHR was independently associated with ultrasound-diagnosed NAFLD, to compare its discriminative performance with that of FBG and HDL-C alone, and to identify patient subgroups in whom this ratio may be most useful.

## 2 Materials and methods

### 2.1 Study population

This study was a secondary analysis of de-identified data originally collected by Yan et al. and deposited in the Dryad repository (17). The parent project was approved by the Ethics Committee of Tongji Medical College, Huazhong University of Science and Technology, and was conducted in accordance with the Declaration of Helsinki. Because the dataset is publicly available and covered by the original ethical approval, no additional institutional review was required for the present analysis.

The parent study recruited adults aged 40–70 years who underwent comprehensive health examinations at Wuhan Union Hospital between January 2020 and November 2021 (18). We applied the same exclusion criteria: excessive alcohol consumption (>210 g/week for men, >140 g/week for women), known chronic liver disease of viral, autoimmune, or drug-related origin, acute illness, renal insufficiency (estimated glomerular filtration rate <60 mL/min/1.73 m^2^), active malignancy, or current use of systemic corticosteroids. Participants with missing biochemical or questionnaire data were also excluded, leaving 1,592 individuals for analysis. This final sample size (*n* = 1,592) was directly inherited from the parent study, as all eligible participants meeting the inclusion criteria were included without additional sampling.

### 2.2 Measurements and definitions

All anthropometric evaluations were carried out by trained staff in a dedicated hospital room equipped for body composition analysis. Participants were required to fast, wear light clothing, and stand on the bioelectrical impedance analyser (BIA) without shoes. A segmented multi-frequency BIA device (Tsinghua Tongfan, BCA-2A, China) was employed to measure body weight, fat mass, and muscle mass, based on the principle of body impedance measurement. The device underwent regular calibration by the manufacturer. Height was recorded concurrently, and body mass index (BMI) was derived as weight divided by squared height (kg/m^2^). The FMR was defined as total fat mass divided by total muscle mass.

Blood pressure (BP) readings were obtained using an electronic monitor (Panasonic, EW3106, China) after the participant had rested in a seated position for at least 10 min. Two measurements were taken at 5-minute intervals, and their average was used for analysis.

All biochemical assays were performed in the hospital's central clinical laboratory. Venous blood specimens were collected following a minimum 8-hour overnight fast to determine platelet count (PLT), total cholesterol (TC), triglycerides (TG), HDL-C, low-density lipoprotein cholesterol (LDL-C), alanine aminotransferase (ALT), aspartate aminotransferase (AST), uric acid (UA), and FBG. PLT was quantified on a Beckman Coulter haematology platform. Serum biochemical parameters were assayed on an AU5800 automated analyser (Beckman Coulter, Brea, CA, USA). TG was determined enzymatically via glycerol phosphate oxidation; TC and LDL-C were measured by enzymatic colorimetric assays using selective detergent solubilisation for LDL-C; HDL-C was quantified by a direct chemical-modification enzymatic approach.

The GHR was computed as FBG (mmol/L) divided by HDL-C (mmol/L). NAFLD was diagnosed according to standard clinical criteria. Hepatic steatosis was assessed by conventional abdominal B-mode ultrasonography (Philips IU22, Philips Healthcare, Inc. N.V.) performed by trained technicians. The diagnosis of fatty liver was based on characteristic ultrasound findings, including increased hepatic echogenicity relative to the renal cortex, attenuation of the ultrasound beam, and impaired visualization of intrahepatic vascular structures and the diaphragm. NAFLD was defined as the presence of hepatic steatosis on ultrasound in the absence of significant alcohol consumption and other causes of chronic liver disease.

Hypertension was defined as either a self-reported history of hypertension or current use of antihypertensive medication. Diabetes was defined as a self-reported history of diabetes or current use of glucose-lowering agents. Tobacco use referred to current or former smoking, and alcohol use referred to current or former drinking.

### 2.3 Statistical analysis

Continuous variables were presented as mean ± standard deviation (SD) or median with interquartile range (IQR), and categorical variables were presented as frequencies with percentages. Baseline characteristics of the study population were compared across quartiles of GHR using analysis of variance (ANOVA) for continuous variables and chi-square tests for categorical variables.

Multivariable logistic regression models were constructed to assess the association between GHR and NAFLD. Three hierarchical models were fitted: Model 1 was unadjusted; Model 2 adjusted for sex, age, tobacco use, alcohol use, hypertension, and diabetes; Model 3 further adjusted for BMI, FMR, PLT, ALT, AST, UA, TC, TG, LDL-C, SBP, and DBP in addition to the variables in Model 2. Covariates were selected *a priori* based on clinical relevance and previously reported associations with NAFLD, rather than by data-driven stepwise selection. Model 2 included core demographic, lifestyle, and comorbidity variables. Model 3 additionally included anthropometric and biochemical parameters that are established NAFLD risk markers or potential confounders of the GHR–NAFLD association. Because GHR was derived from FBG and HDL-C, these two variables were not simultaneously included in Model 3 to avoid structural collinearity and over-adjustment. Multicollinearity among covariates in Model 3 was formally assessed using variance inflation factors (VIF) and tolerance. A VIF >5 was considered indicative of potential multicollinearity, and a VIF >10 was considered severe. Odds ratios (ORs) with 95% confidence intervals (CIs) were calculated. GHR was examined both as a continuous variable (per 1-unit increase) and as categorical variables (quartiles).

Subgroup analyses were performed to examine whether the association between GHR and NAFLD varied across different population subgroups, including sex (female vs. male), tobacco use (no vs. yes), alcohol use (no vs. yes), hypertension (no vs. yes), diabetes (no vs. yes), age (<60 vs. ≥ 60 years), and BMI (<25 vs. ≥ 25 kg/m^2^). All subgroup analyses were conducted using multivariable logistic regression models that included all covariates from Model 3 (i.e., sex, age, tobacco use, alcohol use, hypertension, diabetes, BMI, FMR, PLT, ALT, AST, UA, TC, TG, LDL-C, SBP, and DBP) with the exception of the variable defining the subgroup itself. Interaction terms between GHR and each subgroup variable were introduced into the regression model, and *P* for interaction was calculated.

Receiver operating characteristic (ROC) curves were constructed to evaluate the discriminative performance of GHR for NAFLD. The AUC was calculated for GHR and compared with those of FBG and HDL-C alone using DeLong's test. The optimal cut-off value for GHR was determined based on the maximum Youden index (sensitivity + specificity—1). ROC curves were also constructed for Models 1, 2, and 3 to assess the incremental discriminative value of adding covariates.

All statistical tests were two-tailed, and a *P* value < 0.05 was considered statistically significant. Statistical analyses were performed using SPSS 27.0 and R 4.0.5.

## 3 Results

### 3.1 Baseline characteristics of the study population

A total of 1,592 participants were included in this study. NAFLD was diagnosed in 973 patients (61.1%). Table 1 presents the baseline characteristics of the study population stratified by quartiles of GHR. With increasing GHR quartiles, there were progressive rises in the proportions of males, hypertension, diabetes, and rates of tobacco and alcohol use, whereas the proportion of participants aged ≥60 years declined. Patients in higher quartiles exhibited significantly elevated levels of BMI, ALT, AST, UA, TC, TG, LDL-C, SBP, and DBP, while demonstrating lower HDL-C levels. The prevalence of NAFLD was also markedly higher in the higher GHR quartiles. PLT levels were significantly higher in quartiles 2 and 3 compared with quartile 1 but showed a modest decline in quartile 4. However, no significant differences were observed in FMR levels across the quartile groups (*p* = 0.970).

**Table 1:** Baseline characteristics of the study population by quartiles of GHR.

Variables	Quartile 1 (*n* = 388)	Quartile 2 (*n* = 409)	Quartile 3 (*n* = 397)	Quartile 4 (*n* = 398)	*P*
Male (n, %)					<0.001
Female	155 (39.95)	131 (32.03)	103 (25.94)	55 (13.82)	
Male	233 (60.05)	278 (67.97)	294 (74.06)	343 (86.18)	
Age≥60 years (%)	158 (40.72)	137 (33.50)	121 (30.48)	113 (28.39)	0.002
Tobacco use (%)					<0.001
No	290 (74.74)	277 (67.73)	261 (65.74)	226 (56.78)	
Yes	98 (25.26)	132 (32.27)	136 (34.26)	172 (43.22)	
Alcohol use (%)					<0.001
No	290 (74.74)	287 (70.17)	255 (64.23)	240 (60.30)	
Yes	98 (25.26)	122 (29.83)	142 (35.77)	158 (39.70)	
Hypertension (%)					0.045
No	167 (43.04)	179 (43.77)	164 (41.31)	139 (34.92)	
Yes	221 (56.96)	230 (56.23)	233 (58.69)	259 (65.08)	
Diabetes (%)					<0.001
No	305 (78.61)	323 (78.97)	292 (73.55)	174 (43.72)	
Yes	83 (21.39)	86 (21.03)	105 (26.45)	224 (56.28)	
BMI (kg/m2)	24.35 ± 2.68	25.09 ± 2.79	25.61 ± 2.72	26.57 ± 3.04	<0.001
FMR	0.39 ± 0.12	0.39 ± 0.12	0.40 ± 0.11	0.39 ± 0.11	0.970
PLT (10^9^/L)	205.35 ± 53.60	206.23 ± 50.65	218.33 ± 55.28	214.70 ± 52.73	<0.001
ALT (U/L)	20.21 ± 10.12	23.40 ± 16.46	27.17 ± 20.72	34.06 ± 25.56	<0.001
AST (U/L)	21.72 ± 7.12	21.59 ± 8.48	23.32 ± 12.00	25.88 ± 14.26	<0.001
UA (umol/L)	330.89 ± 83.97	358.50 ± 91.53	383.82 ± 91.36	393.17 ± 103.31	<0.001
FBG (mmol/L)	4.74 ± 0.55	5.08 ± 0.75	5.30 ± 0.81	7.03 ± 2.51	<0.001
TC (mmol/L)	3.87 ± 0.93	4.25 ± 0.94	4.61 ± 0.85	5.17 ± 1.18	<0.001
TG (mmol/L)	0.98 ± 0.46	1.40 ± 0.62	1.96 ± 1.06	3.27 ± 2.67	<0.001
HDL-C (mmol/L)	1.40 ± 0.35	1.15 ± 0.25	1.01 ± 0.19	0.91 ± 0.21	<0.001
LDL-C (mmol/L)	2.10 ± 0.69	2.56 ± 0.77	2.88 ± 0.77	3.11 ± 0.97	<0.001
SBP (mmHg)	126.96 ± 15.12	129.97 ± 16.12	132.18 ± 14.66	134.57 ± 16.34	<0.001
DBP (mmHg)	79.27 ± 10.91	80.11 ± 11.12	82.41 ± 10.82	84.60 ± 11.18	<0.001
NAFLD (%)					<0.001
No	231 (59.54)	183 (44.74)	127 (31.99)	78 (19.60)	
Yes	157 (40.46)	226 (55.26)	270 (68.01)	320 (80.40)	

Data are shown as mean ± standard deviation (SD) or median (IQR) for continuous variables and proportions (%) for categorical variables.

GHR, fasting blood glucose to high-density lipoprotein cholesterol ratio; BMI, body mass index; FMR, fat-to-muscle ratio; PLT, platelet count; ALT, alanine aminotransferase; AST, aspartate aminotransferase; UA, serum uric acid; FBG, fasting blood glucose; TC, total cholesterol; TG, triglycerides; HDL-C, high-density lipoprotein cholesterol; LDL-C, low-density lipoprotein cholesterol; SBP, systolic blood pressure; DBP, diastolic blood pressure; NAFLD, non-alcoholic fatty liver disease.

### 3.2 Association between GHR and NAFLD

Table 2 presents the association between the GHR and NAFLD. In the unadjusted Model 1, a higher GHR was significantly associated with increased NAFLD risk (OR = 5.487, 95% CI 4.099–7.346, *p* < 0.001). This association persisted after sequential adjustment for confounders in Model 2 (OR = 4.262, 95% CI 3.123–5.816, *p* < 0.001) and Model 3 (OR = 1.660, 95% CI 1.023–2.694, *p* = 0.040). When analyzed as quartiles (categorical), a significant positive trend was observed across increasing GHR quartiles (*p* for trend < 0.001 in Models 1 and 2; *p* for trend = 0.017 in Model 3). Compared to the lowest quartile, the unadjusted ORs for NAFLD were 1.817 (95% CI 1.372–2.407, *p* < 0.001) for quartile 2, 3.128 (95% CI 2.335–4.191, *p* < 0.001) for quartile 3, and 6.036 (95% CI 4.384–8.312, *p* < 0.001) for quartile 4. Upon full adjustment (Model 3), the association remained significantly elevated for quartile 3 (OR = 1.733, 95% CI 1.155–2.601, *p* = 0.008) and quartile 4 (OR = 1.741, 95% CI 1.034–2.933, *p* = 0.037), but not for quartile 2 (OR = 1.362, 95% CI 0.969–1.915, *p* = 0.076). Multicollinearity diagnostics for Model 3 showed acceptable tolerance (range 0.132–0.887) and VIF (range 1.127–7.570). The highest VIF was 7.570 for LDL-C, followed by 7.199 for TC; all VIF values were below 10, and the VIF for GHR (quartiles) was 2.351. No severe multicollinearity was observed.

**Table 2:** Association between GHR and NAFLD.

Variable	Model 1		Model 2		Model 3	
	OR (95%CI)	*P*	OR (95%CI)	*P*	OR (95%CI)	*P*
GHR (per 1-unit)	5.487 (4.099–7.346)	<0.001	4.262 (3.123–5.816)	<0.001	1.660 (1.023–2.694)	0.040
GHR (Quartile)						
Quartile 1	Ref		Ref		Ref	
Quartile 2	1.817 (1.372–2.407)	<0.001	1.772 (1.327–2.368)	<0.001	1.362 (0.969–1.915)	0.076
Quartile 3	3.128 (2.335–4.191)	<0.001	2.919 (2.157–3.948)	<0.001	1.733 (1.155–2.601)	0.008
Quartile 4	6.036 (4.384–8.312)	<0.001	4.444 (3.166–6.237)	<0.001	1.741 (1.034–2.933)	0.037
*P* for trend		<0.001		<0.001		0.017

Model 1: unadjusted.

Model 2: sex, age, tobacco use, alcohol use, hypertension and diabetes.

Model 3: adjusted for variables in Model 2 plus body mass index, fat-to-muscle ratio, platelet count, alanine aminotransferase, aspartate aminotransferase, uric acid, total cholesterol, triglycerides, low-density lipoprotein cholesterol, systolic blood pressure and diastolic blood pressure.

GHR, fasting blood glucose to high-density lipoprotein cholesterol ratio; NAFLD, non-alcoholic fatty liver disease; OR, odds ratio; CI, confidence interval.

### 3.3 Subgroup analyses

Subgroup analyses evaluated potential effect modification in the association between GHR (per 1-unit increase) and NAFLD risk (Figure 1). In exploratory subgroup analyses, a borderline interaction with diabetes status was observed (*P* for interaction = 0.046). In non-diabetic individuals, GHR was associated with NAFLD (OR = 2.17, 95% CI: 1.13–4.18, *P* = 0.021). In diabetic individuals, the association did not reach statistical significance (OR = 1.78, 95% CI: 0.82–3.87, *P* = 0.145). However, this differential pattern should not be interpreted as definitive effect modification, given the borderline interaction and the fact that multiple subgroup analyses were performed without adjustment for multiple comparisons. No significant interactions were observed for other subgroup variables, including sex (*P* for interaction = 0.142), tobacco use (*P* for interaction = 0.633), alcohol use (*P* for interaction = 0.661), hypertension (*P* for interaction = 0.617) age (*P* for interaction = 0.398), or BMI (*P* for interaction = 0.169). However, some subgroup-specific associations deserve mention. GHR was significantly associated with NAFLD in females (OR = 4.09, 95% CI: 1.41–11.89, *P* = 0.010) but not in males (OR = 1.34, 95% CI: 0.75–2.38, *P* = 0.323). Similarly, significant associations were observed in non-tobacco users (OR = 2.65, 95% CI: 1.40–5.01, *P* = 0.003), non-alcohol users (OR = 2.41, 95% CI: 1.32–4.43, *P* = 0.004), younger individuals (<60 years, OR = 2.14, 95% CI: 1.12–4.11, *P* = 0.022), and those with higher BMI (≥25 kg/m^2^, OR = 2.72, 95% CI: 1.20–6.18, *P* = 0.017).

**Figure 1:**
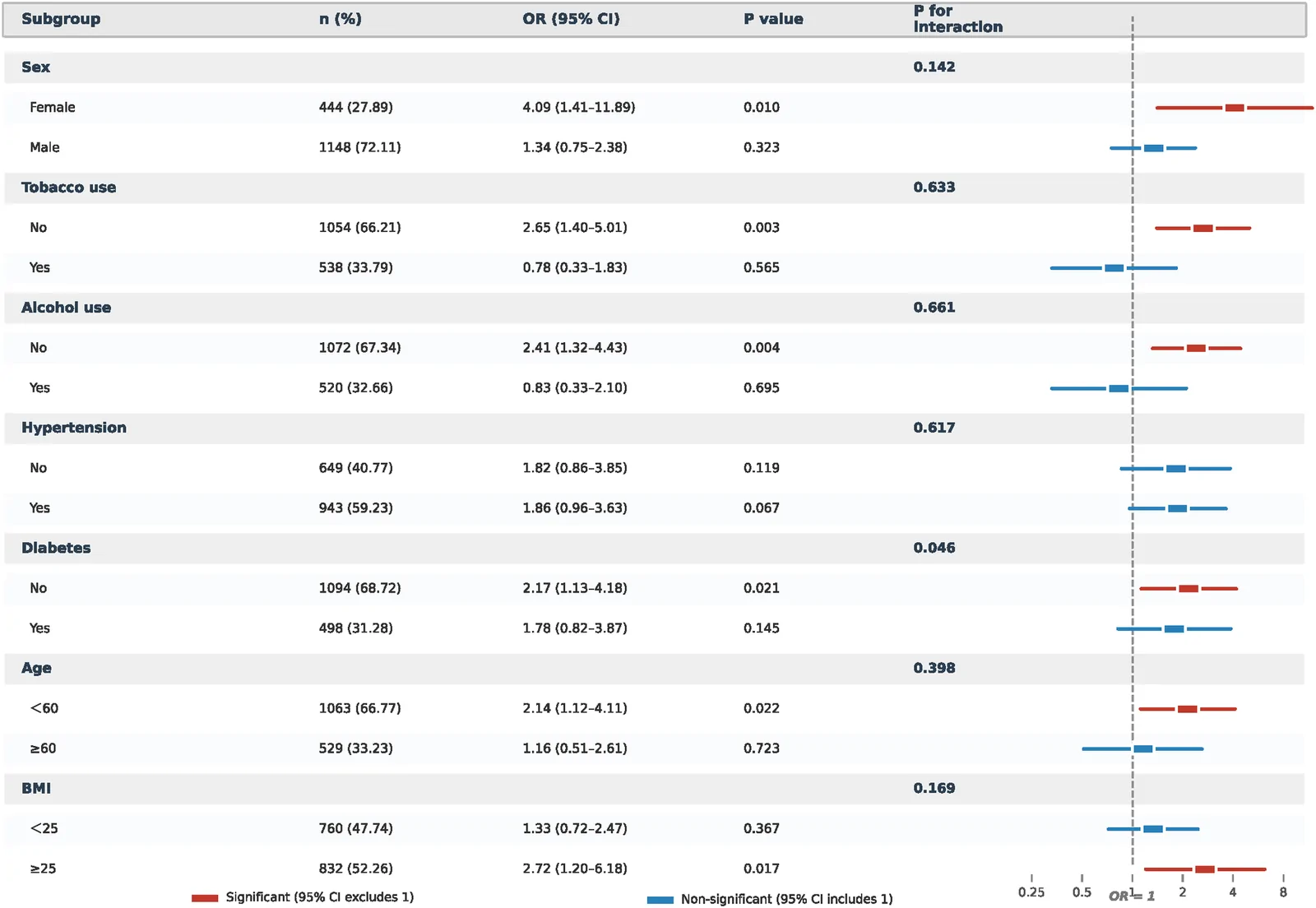
Subgroup analyses for association between GHR and NAFLD. Within each subgroup, multivariable logistic regression models were fitted to estimate the adjusted odds ratios (ORs) and 95% confidence intervals (CIs) for the association between the FBG/HDL-C ratio and NAFLD. All subgroup analyses were adjusted for the same set of covariates as the main multivariable model, including sex, age, tobacco use, alcohol use, hypertension, diabetes, BMI(≥25 vs. <25), fat-to-muscle ratio, platelet count, alanine aminotransferase, aspartate aminotransferase, uric acid, total cholesterol, triglycerides, low-density lipoprotein cholesterol, systolic blood pressure and diastolic blood pressure.

### 3.4 The ability of GHR to predict NAFLD

To evaluate the predictive performance of the GHR for NAFLD in the overall population, we performed ROC analysis and compared it with FBG and HDL-C (Figure 2; Table 3). The area under the curve (AUC) for GHR in the ROC analysis was 0.684 (95% CI: 0.658–0.711), which was considerably higher than that of FBG (AUC = 0.640, 95% CI: 0.612–0.667; *p* = 0.001) and HDL-C (AUC = 0.637, 95% CI: 0.609–0.666; *p* < 0.001). At the optimal GHR cut-off value of 4.482, the sensitivity for identifying NAFLD was 60% and the specificity was 70%.

**Figure 2:**
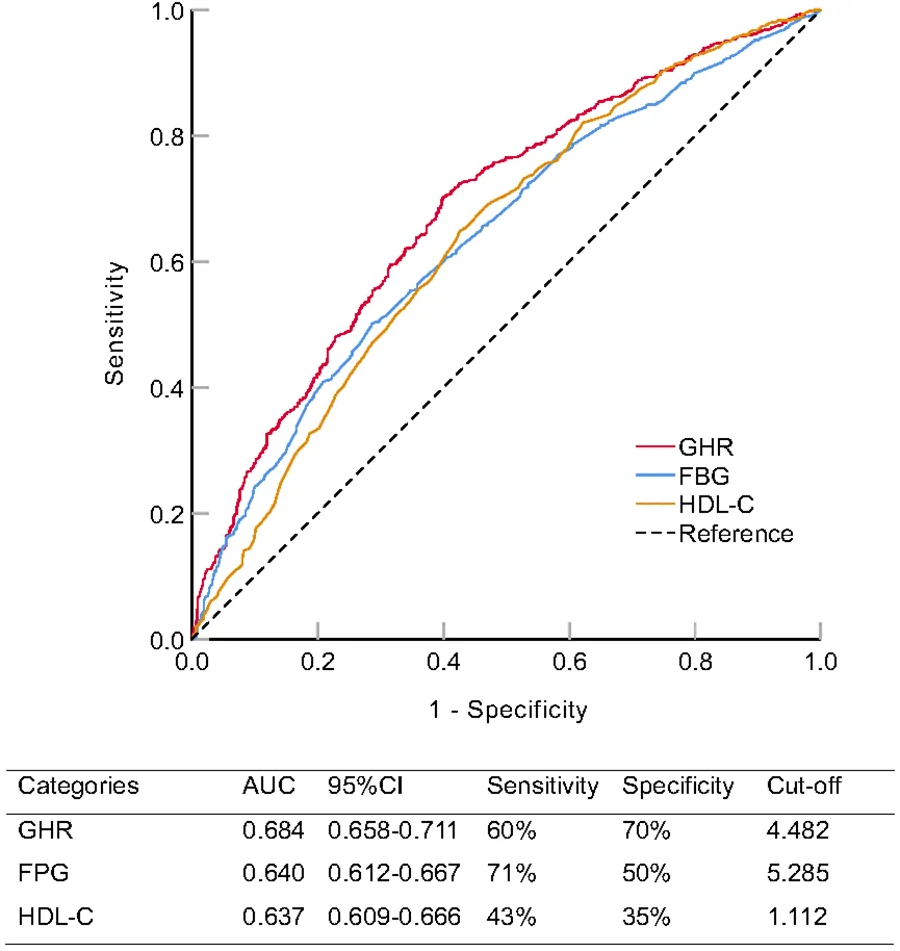
Receiver operating characteristic analysis comparing the discriminative ability of fasting blood glucose to high-density lipoprotein cholesterol ratio (GHR), fasting blood glucose (FBG), and high-density lipoprotein cholesterol (HDL-C) for non-alcoholic fatty liver disease.

**Table 3:** Paired comparison of ROC curves (deLong's test).

Categories	Difference of AUC	Standard error	95%CI	z-value	*P*
GHR vs. FBG	0.045	0.166	0.018–0.071	3.288	0.001
GHR vs. HDL-C	0.047	0.166	0.021–0.073	3.585	<0.001

ROC, receiver operating characteristic; AUC, area under curve; CI, confidence interval; GHR, fasting blood glucose to high-density lipoprotein cholesterol ratio; FBG, fasting blood glucose; HDL-C, high-density lipoprotein cholesterol.

Furthermore, to assess the incremental value of covariate adjustment, we compared the ROC curves across three models (Figure 3). The AUC for the unadjusted GHR (Model 1) was 0.684 (95% CI: 0.658–0.711). After adjustment for demographic and lifestyle factors (Model 2), the AUC increased to 0.714 (95% CI: 0.688–0.740). The fully adjusted model (Model 3) achieved the highest discriminative ability, with an AUC of 0.799 (95% CI: 0.777–0.821), sensitivity of 78%, and specificity of 70%.

**Figure 3:**
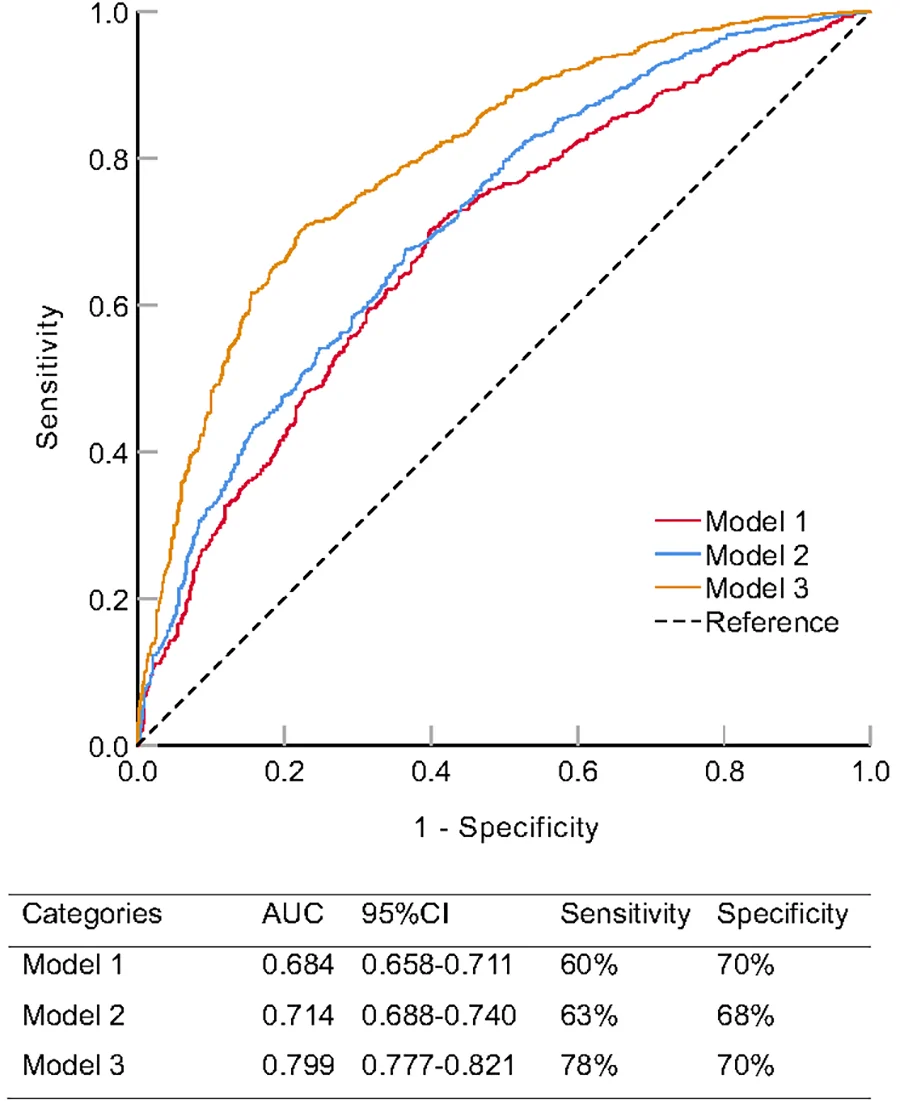
Receiver operating characteristic curves for GHR in relation to NAFLD. Model 1: unadjusted; Model 2: sex, age, tobacco use, alcohol use, hypertension and diabetes; Model 3: adjusted for variables in Model 2 plus body mass index, fat-to-muscle ratio, platelet count, alanine aminotransferase, aspartate aminotransferase, uric acid, total cholesterol, triglycerides, low-density lipoprotein cholesterol, systolic blood pressure and diastolic blood pressure. GHR, fasting blood glucose to high-density lipoprotein cholesterol ratio; NAFLD, non-alcoholic fatty liver disease.

To directly quantify the incremental discriminatory contribution of GHR, we compared the fully adjusted clinical model with versus without GHR. The model without GHR included all Model 3 covariates except GHR. The AUC was 0.798 (95% CI: 0.776–0.820) for the model without GHR and 0.799 (95% CI: 0.777–0.821) for the model with GHR. The *Δ*AUC was 0.001 (DeLong's test, *P* = 0.492), indicating that the addition of GHR did not significantly improve discrimination beyond the other clinical and biochemical variables.

## 4 Discussion

This secondary analysis of a publicly available dataset investigated the association between the GHR and NAFLD. Our findings demonstrated that GHR was independently associated with NAFLD after comprehensive adjustment for a wide range of potential confounders, including sex, age, lifestyle factors, medical history, anthropometric measurements, lipid profiles, liver enzymes, and blood pressure. Furthermore, GHR discriminated NAFLD more effectively than either FBG or HDL-C in isolation, and its incorporation into a multivariable model raised the AUC to nearly 0.80. Importantly, we identified diabetes status as a significant effect modifier of the association between GHR and NAFLD, with a stronger association observed in non-diabetic individuals. Given the cross-sectional design, these findings should be interpreted as evidence of association and cross-sectional discrimination rather than prospective prediction.

In 2023, a multisociety Delphi consensus introduced the term metabolic dysfunction–associated steatotic liver disease (MASLD) to replace NAFLD (19), reflecting the metabolic underpinnings of the disease and avoiding potentially stigmatizing language. In the present study, we retained the term NAFLD because the parent dataset was collected using the original NAFLD diagnostic criteria and does not contain complete information on all five cardiometabolic risk factors required for a formal MASLD definition. Our findings should therefore be interpreted as applying to a population defined by the original NAFLD criteria, and future studies using formal MASLD criteria are needed to confirm these results.

The independent association between GHR and NAFLD observed in our study is consistent with previous reports. Jin et al. using NHANES 2013–2020 data, reported that elevated GHR was independently associated with NAFLD prevalence (OR = 1.22, 95% CI: 1.17–1.28) after adjusting for multiple confounders including race, gender, age, diabetes, BMI, and liver enzymes (14). They also found a positive correlation between GHR and the severity of liver steatosis (*β* = 4.97, 95% CI: 4.28–5.66) (14). Yu et al. first reported in the NAGALA cohort of 14,242 Japanese health examination participants that each unit increase in FHR was associated with a 16% increased NAFLD risk after full multivariable adjustment (OR = 1.16, 95% CI: 1.11–1.21), and identified a nonlinear threshold effect with GHR ≈ 3.0. The GHR significantly outperformed FBG (AUC = 0.71) and HDL-C (AUC = 0.76) alone in identifying NAFLD, with an AUC of 0.79 and an optimal cut-off of 3.89 (20). A subsequent analysis by Xuan et al. using the same NAGALA dataset (*n* = 13,682) further demonstrated that the association between GHR and NASLD was nonlinear and partially mediated by BMI (mediation proportion 59.86%) (15). The magnitude of the association in our fully adjusted model (OR per 1-unit increase = 1.660) is larger than that reported by Jin et al. (OR = 1.22) (14) but comparable to the findings reported from the NAGALA cohort (15, 20). These differences may reflect variations in study populations, adjustment strategies, and NAFLD diagnostic criteria across studies. Of note, GHR has also been linked to atherosclerotic cardiovascular disease in previous studies (21–23), highlighting its broader role as a marker of cardiometabolic risk beyond NAFLD. This distinction is important for clinical interpretation. The metabolic disturbances captured by GHR—insulin resistance and dyslipidemia—are shared pathogenic drivers of both hepatic steatosis and atherosclerosis. Therefore, the association we observed between GHR and NAFLD should be interpreted within this broader cardiometabolic context: GHR does not identify a NAFLD -specific pathway but rather captures an integrated metabolic phenotype that predisposes individuals to both hepatic and cardiovascular outcomes. While GHR may serve as a practical screening tool for NAFLD in health check-up settings, its elevation should also alert clinicians to coexisting or future cardiometabolic risk. Accordingly, our conclusions do not imply that GHR is specific to NAFLD, but rather that it is a useful and accessible indicator of metabolic dysfunction that can aid in early risk stratification for NAFLD.

The biological plausibility of the GHR-NAFLD association is supported by established pathophysiological mechanisms. Insulin resistance is a central driver of NAFLD (8, 9). Elevated FBG is a direct consequence of insulin resistance and impaired glucose homeostasis, reflecting the inability of insulin to suppress hepatic glucose production and promote peripheral glucose uptake (24). Concurrently, decreased HDL-C levels in NAFLD are attributed to multiple mechanisms, including alterations in HDL particle composition, impaired cholesterol efflux capacity, and changes in HDL metabolism (12, 25, 26). In patients with NAFLD, HDL particles exhibit qualitative abnormalities in their lipidome and proteome, with reduced cholesterol content and impaired anti-inflammatory and anti-atherogenic functions (12, 27). Beyond its well-established role in cholesterol efflux, emerging evidence indicates that HDL actively participates in glucose homeostasis (28). Animal and clinical studies have demonstrated that HDL promotes insulin secretion, enhances insulin sensitivity, and facilitates direct glucose uptake by skeletal muscle via the AMP-activated protein kinase pathway (28). By integrating these two metabolic abnormalities, GHR may more comprehensively capture the underlying metabolic dysfunction driving NAFLD pathogenesis. Indeed, glucose metabolic dysfunction and dyslipidemia are closely intertwined in NAFLD, and recent evidence suggests that glucose dysregulation surpasses dyslipidemia in driving hepatic steatosis and fibrosis (29).

Our ROC analysis revealed that GHR (AUC = 0.684) significantly outperformed FBG (AUC = 0.640) and HDL-C (AUC = 0.637) alone in identifying NAFLD. The optimal cut-off value for GHR was identified as 4.482, with a sensitivity of 60% and a specificity of 70%. This finding is consistent with previous studies (14, 15). This superior performance aligns with the dual-pathway interpretation discussed above, wherein GHR integrates both glucotoxic stress and lipid-mediated injury beyond the capacity of either FBG or HDL-C individually. The fully adjusted model achieved an AUC of 0.799; however, this improvement was driven primarily by the other clinical and biochemical variables rather than by GHR itself, as the addition of GHR did not significantly increase the AUC.

Diabetes status showed a borderline interaction with GHR in exploratory subgroup analyses (*P* for interaction = 0.046), with a seemingly stronger association in non-diabetic individuals. However, this finding should be interpreted cautiously because multiple subgroup analyses were performed and no adjustment for multiple comparisons was applied. One plausible explanation is pharmacologic confounding: diabetic individuals frequently receive metformin, statins, or insulin, all of which alter FBG and HDL-C in directions that compress GHR variability and obscure its natural relationship with hepatic steatosis. Importantly, the parent dataset did not contain detailed information on antidiabetic treatment, including drug class, dose, duration, or adherence. Because FBG is a direct component of GHR, glucose-lowering therapy may substantially influence GHR values and attenuate the association observed in diabetic individuals. Another possibility is that diabetic individuals exhibit more severe and complex metabolic disturbances, including profound insulin resistance, chronic hyperglycemia, and advanced dyslipidemia (30). In this context, the incremental contribution of GHR to NAFLD risk may be attenuated, as multiple overlapping metabolic pathways are already strongly activated. Regardless of the underlying mechanism, this exploratory finding may suggest that GHR is potentially more informative in individuals without overt diabetes, where it may serve as a marker of incipient metabolic derangement. However, this hypothesis requires confirmation in prospective studies and should not be regarded as established effect modification.

In exploratory subgroup analyses, the association between GHR and NAFLD appeared stronger in females, non-tobacco users, non-alcohol users, younger individuals (<60 years), and those with higher BMI (≥25 kg/m^2^). However, the interaction tests for these variables were not statistically significant (all *P* for interaction >0.05). Therefore, these subgroup-specific findings should not be interpreted as evidence of effect modification. Sex differences in NAFLD pathophysiology have been described, with some studies suggesting that hormonal factors may influence lipid metabolism and insulin sensitivity differently between sexes (31, 32). The apparent attenuation of the association in males and in older individuals may reflect competing risk factors that become more dominant in these subgroups. The stronger association in individuals with BMI ≥25 kg/m^2^ is consistent with the well-established role of obesity in NAFLD pathogenesis, as excess adiposity amplifies the metabolic consequences of dysglycemia and dyslipidemia (33).

The prevalence of NASLD in our study population was 61.1%, which is notably higher than the global estimate of approximately 32.4% and the reported prevalence of 29.6% among Chinese adults in population-based surveys (1, 4). This discrepancy is primarily attributable to selection bias inherent in our study design. Our participants were recruited from individuals undergoing comprehensive health examinations at a tertiary hospital, a setting that preferentially attracts middle-aged and older adults with pre-existing health concerns or those seeking preventive screening. Consequently, this population exhibited a higher burden of metabolic risk factors—including obesity, dyslipidemia, hypertension, and impaired glucose metabolism—than would be expected in a community-based sample. Furthermore, the age range of our study (40–70 years) represents a demographic with inherently higher metabolic disease risk, whereas population-based estimates often include younger adults with lower disease prevalence. We therefore caution against generalizing our prevalence estimate to the broader Chinese population. Instead, our findings should be interpreted as applicable to health-checkup populations with similar demographic and metabolic characteristics. The primary objective of this study was not to estimate population prevalence but to evaluate the discriminative utility of GHR within a high-risk clinical setting, where early identification and risk stratification are most clinically relevant.

Several limitations of this study should be acknowledged. First, due to the cross-sectional design of the parent study, causality cannot be established. The ROC analysis should be interpreted as an assessment of cross-sectional discrimination rather than prospective prediction, and the optimal GHR cut-off requires external validation. Longitudinal studies are needed to determine whether elevated GHR predicts incident NAFLD and whether changes in GHR over time are associated with disease progression. Second, NAFLD diagnosis was based on ultrasound rather than liver biopsy or magnetic resonance imaging-derived proton density fat fraction. Ultrasound has limited sensitivity for mild hepatic steatosis and is operator-dependent, which may have led to misclassification or underestimation of NAFLD, particularly in participants with mild steatosis. In addition, ultrasound cannot distinguish simple steatosis from steatohepatitis or assess the severity of fibrosis. Although ultrasound is widely used in large-scale epidemiological studies and has acceptable specificity, these limitations should be considered when interpreting our findings. Third, despite comprehensive adjustment for measured confounders, the possibility of residual confounding by unmeasured factors (such as dietary patterns, physical activity, and medication adherence) cannot be excluded. Fourth, information on certain lifestyle factors may be subject to recall bias, although this is unlikely to have systematically influenced the observed associations. Fifth, the study data were collected between January 2020 and November 2021 in Wuhan, a period coinciding with the COVID-19 pandemic. Information on SARS-CoV-2 infection status was not available in the parent dataset; therefore, we could not adjust for its potential influence on FBG, HDL-C, or the derived GHR ratio. Finally, our study population was restricted to Chinese adults aged 40–70 years, which may limit the generalizability of our findings to other ethnic groups, younger or older populations, or non-Chinese geographic regions.

## 5 Conclusion

GHR is independently associated with NAFLD in Chinese adults and offers better discrimination than FBG or HDL-C used separately. Exploratory analyses suggested a possible stronger association in non-diabetic individuals, but this finding requires confirmation. Importantly, adding GHR to the fully adjusted clinical model resulted in only a minimal and statistically non-significant improvement in discrimination. Therefore, the incremental discriminatory value of GHR beyond established clinical and biochemical risk factors is limited, and GHR should not be regarded as a replacement for comprehensive NAFLD risk assessment. Given the cross-sectional design, these findings should be regarded as hypothesis-generating. Prospective studies are needed to determine whether GHR provides any additional clinical utility beyond established risk factors.

## Data Availability

The datasets presented in this study can be found in online repositories. The names of the repository/repositories and accession number(s) can be found below: https://datadryad.org/dataset/doi:10.5061/dryad.7d7wm3809.
